# How health professionals conceive and construct interprofessional practice in rural settings: a qualitative study

**DOI:** 10.1186/1472-6963-13-500

**Published:** 2013-12-01

**Authors:** Vicki Parker, Karen McNeil, Isabel Higgins, Rebecca Mitchell, Penelope Paliadelis, Michelle Giles, Glenda Parmenter

**Affiliations:** 1 School of Health, University of New England, Armidale, NSW, Australia; 2 Hunter New England Nursing and Midwifery Research Centre, Hunter New England Area Health District, Newcastle, NSW 2300, Australia; 3 Newcastle Business School, University of Newcastle, Newcastle, NSW, Australia; 4 School of Nursing, University of Newcastle, Newcastle, NSW, Australia

**Keywords:** Interprofessional practice, Rural contexts, Qualitative methods, Health professionals

## Abstract

**Background:**

Although interprofessional practice (IPP) offers the potential to enhance rural health services and provide support to rural clinicians, IPP may itself be problematic due to workforce limitations and service fragmentation. Differing socioeconomic and geographic characteristics of rural communities means that the way that IPP occurs in rural contexts will necessarily differ from that occurring in metropolitan contexts. The aim of this study was to investigate the factors contributing to effective IPP in rural contexts, to examine how IPP happens and to identify barriers and enablers.

**Methods:**

Using Realistic Evaluation as a framework, semi-structured interviews were conducted with health professionals in a range of rural healthcare contexts in NSW, Australia. Independent thematic analysis was undertaken by individual research team members, which was then integrated through consensus to achieve a qualitative description of rural IPP practice.

**Results:**

There was clear evidence of diversity and complexity associated with IPP in the rural settings that was supported by descriptions of collaborative integrated practice. There were instances where IPP doesn’t and could happen. There were a number of characteristics identified that significantly impacted on IPP including the presence of a shared philosophical position and valuing of IPP and recognition of the benefits, funding to support IPP, pivotal roles, proximity and workforce resources.

**Conclusions:**

The nature of IPP in rural contexts is diverse and determined by a number of critical factors. This study goes some of the way towards unravelling the complexity of IPP in rural contexts, highlighting the strong motivating factors that drive IPP. However, it has also identified significant structural and relational barriers related to workload, workforce, entrenched hierarchies and ways of working and service fragmentation. Further research is required to explicate the mechanisms that drive successful IPP across a range of diverse rural contexts in order to inform the implementation of robust flexible strategies that will support sustainable models of rural IPP.

## Background

Approximately half the global population lives in rural areas [1] where residents have higher rates of chronic disease, injury and early death compared with people living in metropolitan areas [2]. There are also major health workforce shortages in rural areas along with poor access for rural residents to a range of health-care services [3]. The health workforce shortage in rural areas has far-reaching implications for how health workers practise with major differences in work practice and scope between metropolitan and rural clinicians.

Rural health practice is distinguished by more generalist approaches to healthcare and service models which differ from those found in metropolitan centres [4]. Patients are faced with the struggle of negotiating a fragmented health system where there is a historical “‘disconnect’ between general practice, acute care and community health services” [5], p. 85]. Moreover, health professionals working in rural settings are likely to provide a broader range of services, work longer hours, operate without adequate locum coverage, have restricted access to specialist expertise and have limited access to professional support networks [6]. Professional boundaries are often less clear, with a need for multiskilling and flexibility in accordance with limited resources and other constraints [7]. In contrast, metropolitan practice is generally more specialised with a diverse and large workforce with defined discipline boundaries and scope within with a wider range of services, and resources than is available to rural practice [8].

Interprofessional practice (IPP), defined as teams of professionals with diverse skills working together synergistically to achieve optimal outcomes for patients and their families [9], has been promoted as a key factor in improving the effectiveness of health services in a number of countries [10-12] particularly in rural and remote areas [6,13]. While there is some evidence to suggest that IPP teams provide a more clinically effective service, generate better health outcomes, are more innovative and patient-focused [11,14], other studies have demonstrated that interprofessional collaboration can be hampered by communication barriers, power and status differences, and a lack of knowledge other health profession’s roles and expertise [15-17].

Nonetheless, the implementation of IPP has been associated with positive healthcare and professional outcomes in rural settings. Integrated IPP service provision in rural areas has been found to improve patient care, satisfaction with care, enhance cost-effectiveness and provider learning [18,19]. IPP work has also been linked to increased job satisfaction and retention in rural areas [20,21]. There is also evidence that IPP teams enhance professional development across health specialties and alleviate professional isolation [22]. However, according to Bourke, Coffin, Taylor, & Fuller [23] there have been limited reports of success in achieving true IPP in rural contexts “with most rural health, practitioners and academics alike, work within their own disciplinary boundaries. Communication and shared language between disciplines and cultures are lacking” (p. 5).

Whilst offering potential to enhance services and overcome some of the challenges faced by rural clinicians [24], IPP may itself be problematic due to the reduced number of health care workers across a small number of professions. Differing socioeconomic and geographic characteristics of rural communities means that the way that IPP occurs in rural contexts will necessarily differ from that occurring in metropolitan contexts. Furthermore, while the Australian healthcare system and context is unique, very similar issues occur in rural health in Canada, United States, New Zealand, United Kingdom and parts of Europe [25]**.**

## Methods

### Research aim and relevance

This study’s aim was to investigate the factors contributing to effective IPP in rural contexts, to examine how IPP happens and to identify barriers and enablers.

### Design

The study was guided by a qualitative descriptive approach using Realistic Evaluation [26] as a research framework. This approach asks, what works for whom in what circumstances? In this study it encompassed policy, organisational and management influences in rural interprofessional environments and explored the participant perceptions about supportive mechanisms as well as expected and observed outcomes [27]. Interviews were used to gather in depth information from individual managers and clinicians. Interviews were conducted rather than focus groups because of the logistical difficulties of getting clinicians together due to distance and workforce shortages.

### Recruitment

Invitations to participate were distributed to eligible rural health sites. Participants were purposively recruited to ensure representation of professions and role functions, including managers and policy makers, across a range of regional and rural geographic settings, across sectors and types of health care facilities i.e. community health centres, hospitals, individual practices and multi-purpose services. The professions, roles and settings of participating health professionals are detailed in Table 1.

**Table 1:** Summary of participating health professionals

**Health profession/role**	**Number**	**Setting**	**Number**
Health service manager^1^	7	Acute	14
Medical officer	3	Community	16
Nurse manager	2	Primary	3
Registered nurse	5		
Allied health practitioner (AHP)	3		
Clinical nurse consultant	2		
**TOTAL**	22	**TOTAL**	33^2^

^1^Health Service Managers had professional backgrounds in either nursing or allied health.

^2^Nine participants worked across two settings, one participant worked in three settings.

### Data collection

Data collection comprised semi-structured interviews with 22 health professionals over a period of twelve months in 2011 and 2012. In line with Pawson and Tilley’s [26] framework, clinicians were asked about their experiences of and professional responses to interprofessional work, the barriers to such collaboration, and facilitating factors. Managers and policy makers were asked about the role of policies, resourcing and structural influences, and the extent to which interprofessional approaches exist at organizational and institutional level. Interviews began by asking participants about their experiences and views of IPP in their own situation and were structured around the following questions:

• How does IPP happen? Who is involved, when, and why, what decision making occurs, what outcomes ensue.

• Under what circumstances is IPP most effective?

• What barriers exist to successful IPP?

• What changes are required to make IPP more effective?

Interviews lasted between 20 minutes and 90 minutes and were transcribed later for analysis by the research team.

### Ethical considerations

This study was approved by an accredited NSW Health Department ethics committee (HNEHREC 10/06/16/4.01). Informed written consent was obtained through the delivery of an information statement written in plain language which outlined the purpose of the study. Participation in the study was entirely voluntary and interviewees were given the option of withdrawing from the project at any time without giving a reason.

In order to maintain confidentiality of participant information and comments, interviewees were assigned code numbers and these codes were used throughout the research process. To protect the anonymity of informants, very limited demographic information has been included in the results. This is essential given the close-knit nature of the rural communities studied.

### Data analysis

Interview transcripts were read by all research team members. Researchers independently coded, collated and inductively derived categories and themes from the data, specifying their relevance, dimensions and parameters. Research team members then shared and discussed their collective findings which were then rationalised and consolidated. Finally, these endorsed themes were worked into a comprehensive description, populated with quotes to ensure grounding in the data and representation across participants to provide an integrated account of participants’ views and experiences of IPP. This textual representation was validated by the full research team.

### Trustworthiness of the research

In keeping with requirements for qualitative research, trustworthiness is demonstrated through reference to credibility, confirmability, dependability and transferability [28]. To this end, rigour was ensured through independent researchers analysing the data and then comparing across researchers for consensus, by keeping an audit trail of activity linking summary data and interpretations to original source material and by adhering to consistent and ethical research processes. The potential for transferability is achieved by providing:

… sufficient detail of the context of the fieldwork for a reader to be able to decide whether the prevailing environment is similar to another situation with which he or she is familiar and whether the findings can justifiably be applied to the other setting [28], p. 63].

## Results

The study findings are reported in two sections, views and experiences of IPP reported by study participants and enablers and barriers to rural IPP.

### Participants’ views about and experiences of IPP

#### Valuing of IPP

Across all participants it was a taken-for-granted that IPP was a good thing and that it is instrumental in achieving quality healthcare and beneficial outcomes for patients**.** Although there were many reasons why IPP was seen as important, such as support for and learning from each other, shared problem solving and rationalisation of effort, the most cited benefit was improved access and care for patients.

And you aren’t overly reliant on a one to one type relationship…There’s learning between different health professionals I think, sharing information, it value adds to the care (Medical General Practitioner (GP).

I think there are a lot of benefits from different professions working together as far as the continuity of care for patients and I think also a more holistic look at how patients are managed, because if the different professions are speaking to each other and talking to each other all the time then you’re getting a more rounded view of the patient and what the issues are (Nurse Manager).

In spite of the universal acceptance of IPP, there were disparate views about what IPP is, whether it actually occurs and varied descriptions of how it occurs. Some participants were unequivocal that IPP was a feature of their practice, for example,

I see interprofessional practice is what we do, what I do every day (Medical Officer) (MO).

The following comment from a Health Service Manager (HSM) sums up the view of most participants that a comprehensive approach to care requires a team approach:

Because we’re dealing with not just one particular issue or not just one particular concept, and because you’re dealing with health and health is influenced by so many different things, naturally, you’re taking a comprehensive approach and if you’re taking a comprehensive approach you need participation of everybody in the team. You never do anything on your own. You just can’t do things on your own. You can’t function in a silo.

#### IPP as complex and varied

All participants recognised the significance of working with and across disciplines and indicated ways in which they were participating in IPP. How they varied was in the purpose of their engagement and the level at which they were willing and able to invest, ranging from direct care contexts and education of patients and staff to policy development and whole of community health service planning and provision. It is clear that ways of working together vary according to each peculiar context and availability of health services and that IPP is complex and operates in different ways to inform and achieve different agendas and outcomes.

*You do it differently because of circumstances and the context is different. Generally, again, it comes down to that recruitment and retention and having the availability of that interprofessional team. You might have a dietician but it’s only limited hours, so it makes it more challenging (AHP)*.

Generally reports of IPP fell into the following broad forms:

**Routine meetings** include those activities that are planned and organised such as interprofessional team meetings. These usually occur on specific hospital wards or units, however their success depends on participation of all members, which is not always the case:

We haven’t had much luck in getting GPs to case conferences as you can imagine, it’s usually a really complex case that involves lots of other organisations where we can manage to get a GP involved, which is you know a bit sad but that happens (Nurse Manager).

**Ad hoc case conferencing** was identified as occurring for three reasons; for problem solving complex intractable clinical problems, where health service utilization is high or for policy implementation:

… you encourage people to work together in order to solve a problem or in order to work together to help a client. You may call a case conference, or you may form a working party, in order to work on a policy directive… Also if there’s a client that may be using a lot of service providers within community health, we might have a case conference just so everyone knows what the other party’s doing, so we’re not overlapping with referrals and that sort of thing (HSM).

**Referral** also occurs where a clinician usually GP, service manager or discharge planner refers to one or more other clinicians. Referral may or may not include a requirement for or commitment to ensuring feedback. Referral and sharing of clients occurs across professions, services, health care sectors, specialist and generalist services and rural and metropolitan service providers. It occurs in formal and informal ways.

Others suggested that what occurs is not IPP at all, but simply a range of practitioners who ‘use’ each other’s services (**sequential care**), most often through referral processes. In this way patients are handed over at particular points in their health care journey rather than their care being designed and delivered through shared decision making and planning. It is not that practitioners do not believe in the value of IPP but they see that the opportunity for true interprofessional working is limited by workload constraints and adherence to certain ways of working. This was the case particularly for Allied Health practitioners working in the community, as one participant explained:

for community patients there is very limited opportunity for us to work interprofessionally because we may be working with the same patient but we’re picking them up at different times (AHP).

Interprofessional consultations, sharing of information and handover of clients also occurs **serendipitously** through corridor conversations as suggested below

But it’s not a set planned thing and I guess that happens all up and down the corridor in our offices because we have sort of an open-door vibe here. People do just walk in and say, “So what’s happening with such and such?” And you can very quickly get a rundown on where the care is up to and if they need anything new or that kind of thing (AHP).

At times IPP was considered to be the result of shared understanding and planning of **integrated services**. Instances of integrated care were described where there is continuous involvement of various professionals with feedback and shared decision making, usually incorporating broader functions such as education, social support, together with involvement of patients and families. These practices were identified as occurring in palliative care, rehabilitation, transitional aged care, Aboriginal services and some child and family services. Decisions about care provision and who is best placed to provide care are often complex, particularly for clients with chronic disease or cancer.

### Enablers and barriers of rural IPP

In many instances factors that were seen to impact IPP were identified as operating to either enhance or impede IPP, for example workload and time constraints. Specific enablers of IPP were identified as: belonging and connection to community; individuals who were able to engage and connect services; formal and informal communication strategies; funding models, in particular the Australian government health insurance (Medicare) rebates for Enhanced Care/Chronic Care Programs; co-location of services and excessive workload. Barriers identified included workload and workforce limitations; non-valuing of the team or other health professionals; and absence or fragmentation of services.

### Enablers

#### Connection to community

In the main, rural health care is provided by health professionals who are members of the local community. This means that they have local knowledge of the place, its people and the socioeconomic and historical circumstances that impact on the town and the health of the community. This connection to place, people and purpose means that local health professionals often share the same concerns and the same challenges. They also quite often share the same patients.

There is a strong community connection. I also think most of us have got a (shared) vested interest in our communities (HSM).

I’ve got such good local knowledge. You know the people who come into hospital, you know their carers, you know where they live and that’s the beauty really of living in the country. Even though you can be isolated and marginalised as far as getting services or getting people specialist treatment, they’re the benefits because you know people on a more intimate level. So you’re fortunate in the fact that you’ve got a more hands on approach to following up with people (Registered Nurse (RN)).

This history of shared experience has meant that participants see what they do as inherently interprofessional, which in their view makes IPP more important and more likely to succeed. They also believe that it is logistically and geographically easier for them to engage in IPP than it is for their metropolitan counterparts.

Because it is a small town, the people we are working with are generally friends. So we’ve got a good social relationship as well as a professional relationship. So I certainly think there’s more benefits to working in the country in this sort of respect with, knowing the people you’re working with so you’re able to talk to them. You’re not as standoffish about approaching someone to ask advice or ask for referral and that sort of thing (RN).

#### *Pivotal roles*

Participants identified a number of key roles which were critical in championing, initiating or maintaining IPP within their domain or across healthcare settings. The role of the GP is critical in rural healthcare. As often the first point of contact for patients GPs contribute to IPP in a number of ways; through co-ordination of Medicare funded packages, in collaboration with Practice Nurses, and through employing various professionals within their practices, or by co-opting professionals to run or participate in clinics. They also participate to varying degrees in Multidisciplinary care and team meetings in MPSs and hospitals.

And I guess it’s even more apparent since Medicare funded all of these care plans so that allied health practitioners can now access Medicare in certain circumstances, and GP’s have kind of become the gatekeeper of chronic disease management, I suppose. So I am continually referring patients to allied health practitioners and then they’re continually communicating back with me (MO).

Along with the GP, other professionals who played pivotal roles, initiating and co-ordinating interprofessional engagement, included service managers in community health, Practice Nurses and discharge planners in hospitals.

… [the Discharge Planner] she’s sort of the glue… that holds us all together because she’s got this extensive knowledge and extensive contact base for all of it really: the residential aged care facilities, your HACC [Home and Community Care] services, anybody and everybody that’s involved in that external relationship, she’s the sort of pivotal point… Although she’s line managed by the acute service, by myself, she crosses over evenly really across all of those internal and external disciplines. She’s the key (Nurse Manager).

Participants also recounted examples of where Practice Nurses had become the principle point of contact for the coordination of care, preparation of health care plans for those with chronic illnesses and recruitment of patients to participate in programs while doing immunisations.

#### *Funding*

Some of our participants discussed how interprofessional collaboration between GPs and other health professionals has been fostered via government health insurance rebates for referrals to AHPs under Enhanced Care/Chronic Care Programs:

I think since the introduction of the fact that allied health practitioners can now access Medicare in certain ways that’s actually precipitated an increase in that sort of communal management of people…So it’s the introduction of the Care Plans, I would have to say (MO).

One interviewee described how funding opportunities can also drive practitioners to collaborate across health settings:

Also have been able to share employees and capitalise on funding… And so on a monthly basis I actually meet with the chief executive of the Medicare Local now and so we've been meeting for over six years on a monthly basis and we discuss programs (Health Service Manager).

#### Proximity and colocation

Another way in which IPP is made possible in rural areas is through bringing professionals together in the one site, usually within a GP Practice or MPS. This enables patients to see a range of professionals without the need for extensive and burdensome travel. This model is not only effective in creating interprofessional teams but it also ensures timely consultation with necessary services. Participants reported that prior to introduction of these models some patients were waiting up to eighteen months for professional services, often having to travel two and half hours to a major centre. Having a range of services within a practice or MPS also allows patients to be engaged more effectively in their own care, especially through increased opportunity for education. This is achieved through funded care plans for patients with a chronic disease.

…It just reinforces and helps I guess the patients to begin to be part, own their care and it reinforces what you can offer in a short time…we have a diabetes clinic within our surgery and we have an Educator and a Dietician who come to the surgery. And the reason why we did that was it was taking up to a year to 18 months for some patients to actually access care through the diabetes clinic [in a larger centre], so you know it was just “mission impossible” trying to fit people in. So the way that works is through the Co-ordinated Care Management plans and then through Medicare (MO).

Participants also recounted examples of where colocation of health practitioners promoted referral and sharing of clients in formal and informal ways:

We’re all, we’re quite informal with most of our liaison with the other professionals because pretty much all [the team] is up on this floor and so we can simply walk down to someone else’s office and you sit down and just have a chat with them about what’s going on…(RN)

Because I share an office with an occupational therapist there’s a lot of informal conversations about cases that obviously are relevant to both of us (AHP).

#### Workload and workforce drivers

Some participants explained that IPP exists out of necessity and is driven by excessive workloads and lack of workforce. This was the case particularly for Allied Health Professionals (AHP).

I guess we probably don’t do as much active intentional interprofessional… But I guess that’s probably to do with workloads and those kinds of issues. But there’s definitely a lot of interaction between different professions in our team (AHP).

Because they are few in number and each some of them, particularly AHPs, may be likely to be the only member of their profession in town, rural health professionals have become highly reliant on each other for advice, support and to share the load.

…[the] allied health team and other health workers are your support network and your team as well (AHP).

It’s most effective for me because you’re sometimes in these sorts of positions you can feel like you’re a sole practitioner, you feel like you’re making all the decisions yourself…what’s been most effective for me is gathering in all these other people around me and all working together and not feeling like you’re working alone (RN).

This level of interprofessional support was clearly demonstrated by examples of team members providing support for overstretched colleagues. Working in an interprofessional team also conveyed additional benefits in terms of professional development and learning to appreciate different disciplinary perspectives:

where I’ve had more to do with Allied Health, it’s taken me a while but I realise that they’ve actually got a totally different mindset or they’re taught a different way of looking at patients than nurses do, so I think that’s a really good thing to bring to a case discussion about clients (NM 1).

### Barriers to IPP

#### Workload and workforce limitations

Whilst excessive workload was cited as a driver of IPP, it was most often viewed as an impediment to interprofessional working. In many of the study sites there were minimal numbers of health professionals representing a small number of disciplines working across a large geographic area. There could be no-one, or very few people with whom to share information and consult with about patient care. This was the case particularly for Allied Health practitioners working in the community, as one participant explained:

Most of the clinicians on staff are extremely busy… because we have waiting lists and different prioritisation schedules and tools in terms of how we prioritise our patients, it’s very hard to pick up the same patient at the same time (AHP).

It would be great if we had a dietician because a lot of my work goes hand in hand with them. And with having a very, very limited service, the most interaction I get with her is basically just email (AHP).

It was felt by some that excessive workload over protracted periods of time meant that staff were overburdened and often too tired to consider how they might engage in a more effective way.

The barriers are that for all staff the doctors and the nursing staff and Allied Health is their workload, they certainly do struggle sometimes with their workload. And I guess the other barrier is when people put themselves before what we’re trying to achieve, and that could well be related to their workload as well. I think more often than not the workload and with that tiredness comes an inability to be able to see the forest for the trees (HSM).

#### Non valuing of the team and its members

Participants recounted numerous instances where IPP was hampered by professionals not knowing each other’s roles, not being considerate of or communicating effectively with other team members. This was believed in part due to entrenched traditional hierarchies and ways of working. GPs can be pivotal in driving IPP, they were also identified by some participants as at times not being willing or able to participate effectively with the IPP team. This was recognised by a variety of members across teams, including doctors themselves.

I guess, by a lot of history, doctors have got a very specific place in the health hierarchy and many of them. I won’t say play on it but they think that they are at the top of the pinnacle and they don’t always like to take other people’s views into consideration (MO).

*Barriers are when people don’t want to be game players with the larger team. So if you’ve got a client’s GP who sees the client on a regular basis but they don*’*t give you feedback*, *but they complain when you don*’*t give them feedback. So they just do their own thing and they*’*re not ensuring that they are part of the larger network and ensuring that other people in the treating team know what they*’*re doing* (*Clinical Nurse Consultant* (*CNC*)).

There was also recognition that some clinicians don’t readily engage in IPP and that it takes time to build the conditions and processes necessary to develop knowledge and trust in each other’s skills.

*There are some personalities that just don*'*t*, *really feel comfortable in terms of engaging in that model. And so it takes time to do that and knowing each other*’*s kind of skills*. (*MO*)

…*and the other thing is actually making sure that we all understand everyone else*’*s role. That*’*s actually really important*…*I think it*’*s something every health professional should understand*, *that whole health care team and who does what*, *where and when*, *to be able to support your clients the best you need to. But certainly in rural practice it*’*s knowing who that person*, *that one person is to contact* (*AHP*).

#### Absence of and fragmentation of services

A number of managers and nurses with area wide jurisdiction pointed out the complex and often fragmented way in which IPP occurs. There was a strong view that although numerous mechanisms for IPP exist at a range of levels across sites and contexts, often these mechanisms do not connect or inform each other. As one CNC said:

*There are three separate multidisciplinary team discussions that I*’*m aware of that have different structures and different outcomes attached to them. And I don*’*t even know really what goes on in discussion in the community health and in GP practices and whatever else*.

Despite the recognition by GPs that IPP is increasingly required to treat patients with complex chronic conditions and co-morbidities, links between GPs and other community providers are reported as limited. Furthermore, we found some evidence of gaps in communication processes between AHPs and GPs:

…*For some of the allied health stuff it*’*s sometimes seems a bit amorphous*…*For example*, *you send someone for podiatry*, *and you*’*ll get an initial thing back and they*’*ll have done a very good assessment*, *but it kind of then disappears into the ether*… (*MO*).

Different models of IPP exist because of different funding programs for different types of illnesses, differing contexts with varying available services and staff and the specific interests and skills of individuals. Many models are fragile in that they depend on the continued availability of one or two health professionals.

*We used to have a child development clinic that has fallen by the wayside with workload and change of staff and recruiting vacant positions and things like that. So hopefully it will come back in time but it was just for*…*the first three years of life*, *if the parent was concerned*, *to bring them in and be able to see three allied health staff and a community child and family health nurse in the one room and have that kind of one*-*stop shop situation* (*AHP*).

### Overcoming barriers

Participants also suggested ways in which some of these perceived barriers could be managed. For example, along with the need for adequate numbers of professionals successful IPP requires the development of a culture of open and critical engagement, sharing and safety, directed towards patients and their care. In order for this to happen there is a:

*Need to define roles & responsibilities*; *provide a safe environment for open communication. It really comes down to the professionals themselves and their willingness to actually look at interprofessional practice*, *where people can feel free to say and critique what*’*s happening with that patient* (*MO*).

Another Medical Officer highlighted how the lack of shared language, history and education could be remedied by interprofessional education efforts which focussed on role understanding:

*I think that if we still educate people in silos*, *if they continue to be educated in silos then you will still have this kind of arrogance between professions that need not be there*…*But I do think that if we can get the students to have some perception of what the roles are of these other people and respect them and then that*’*s heading in the right direction* (*MO*).

## Discussion

The aim was investigate to the factors contributing to effective IPP in rural contexts, to examine how IPP occurs in rural contexts, and to identify barriers and enablers. There was clear evidence of IPP in the rural settings where this study was conducted that was supported by many descriptions of collaborative and integrated practice. There were also instances where IPP doesn’t and could happen. This uneven implementation of IPP within our study is consistent with the mixed results of IPP found in the literature [23,29]. In spite of the diversity and complexity of IPP in rural contexts there were a number of characteristics identified that significantly impacted on IPP. These were: the strong community connection and the history of shared experience; health professionals with authority and opportunity to initiate processes that engage others; funding to support IPP; proximity and colocation; workload and workforce limitations; the presence of a shared philosophical position characterised by recognition of the benefits of IPP and valuing of and respect for others; and absence and fragmentation of health services.

Community connection and local knowledge plays a key role in rural health service provision. For instance, nurses have been described as the “'agents of connectivity'…providing essential linkages between the system's many users, health professionals and service arrangements” [24]. Rural nurses in general have described ‘knowing’ their local community as a positive characteristic of their role and this enables them to facilitate links between local health providers and advise patients on available community resources [30,31]. This was reinforced in our data, particularly with the hospital-based Discharge Planner who indicated that good local knowledge informed care plans as well as follow-up.

This highlights the importance of professional roles that span boundaries and facilitate communication across sectors. The Discharge Planner strengthened ties and communication between acute and community services. In addition, GPs in primary care were pivotal in engaging other health professionals in coordinated care for those patients with chronic conditions. Gittell [32] describes these roles as “boundary spanners” – individuals who cross functional or organizational boundaries in order to integrate or link the work of other care providers.

GPs are also pivotal in integrating care across the primary and acute care settings in rural areas as they generally have existing connections with local hospitals [33]. Although referrals from GPs to other health professionals have been supported by Commonwealth government rebates under the Enhanced Care/Chronic Care Programs [34], there is evidence to indicate that having the GP as the pivot or care coordinator is not without problems. Collaboration between GPs and other health care providers have been marred by imprecise and contradictory role definitions [35], mistrust and perceived threats to autonomy and independence [36]. In addition GPs have a history of referring patients to other health professionals in an inconsistent and uncoordinated manner [37]. A number of participants (including a medical officer) discussed barriers associated with the attitude of the medical profession to IPP. Some of the associated issues included lack of awareness of how other professions can contribute to decision making, difficulties in engaging doctors in the process as well as the perceptions of medicine’s place in the health hierarchy. Additionally, our study also revealed some fragmentation of IPP mechanisms across sites and contexts. Spanning organizational boundaries in the delivery of health care confounds IPP as the boundaries between services, roles and professional groups are changing and this adds to uncertainty and the vulnerability of those involved [36].

Funding arrangements for health care in rural areas impact significantly on the potential for IPP. Primary health services in Australia are delivered via a complex mix of private providers, state government-funded health services and fee-for-service arrangements supported by Commonwealth funding [34]. Linkages between GPs and other health professionals have been promoted via government funding for Practice Nurses and Medicare rebates for referrals to AHPs under Enhanced Care/Chronic Care Programs [34,38]. Integration of primary health care services (such as MPSs) has also been funded by various decentralized initiatives funded by both state and Commonwealth governments [34]. Our data supports the evidence that collaboration between GPs and other health professionals has been boosted by government funding and additional Medicare rebates.

Co-location of health providers fosters collaboration, is likely to provide the greatest benefit to those suffering chronic illness [34,39] and has been viewed as a key factor in sustaining IPP in a range of settings [21,34,40]. In our study, co-location was seen as particularly beneficial in facilitating informal discussion and review between practitioners and providing integrated services in a GP practice or MPS for those with chronic illness. Co-location of services alone, however, does not necessarily guarantee integration of services.

Rural health services face substantial challenges in recruiting and retaining adequate numbers of health professionals [24]. Such workforce shortages mean that rural practitioners struggle with problems of inadequate locum coverage, limited professional support networks and excessive workloads [6,7]. For some of our participants, workforce shortages and extended vacancies in particular disciplines made IPP challenging. Furthermore, heavy workloads can place undue stress on clinicians and hamper their readiness to engage in IPP. Yet in other instances, heavy workloads became a driver for clinicians to work interprofessionally. This supports the view that collaboration and teamwork in rural practice are influenced by workforce limitations and the “consequent need to work cooperatively to ‘get the job done’” [41], p. 145].

The reduced number of health professionals means that clinicians are often working alone or as solo practitioners in a small team [42]. Our study presents evidence of how professional isolation can be alleviated via teamwork and successful IPP. Nursing staff, managers and AHPs consistently expressed how interprofessional teams offered professional support as well as provided them with a strong sense that they were not managing alone. Such findings support an earlier commentary that in comparison to urban teams, there appears to be greater respect for the work of different professions in rural and remote practice [43].

Change is occurring in the ways rural professionals engage with each other and how their relationships inform models of care for people with varying health problems. Funding models are driving change through funding linked to joined-up care, recognising the need for transition and the potential for gaps across sectors. The difficulties confronting professionals and the IPP agenda are complex and often historically embedded.

To achieve optimum IPP outcomes there is a need for cultural change, trust, respect and sharing of information and communication across professionals. Mutual respect and shared values along with an knowledge of the roles and responsibilities of other care providers have been noted as key competencies for interprofessional working [44]. These elements can be fostered by clinicians sharing information and learning from one another during practice as well as by interprofessional education efforts [44]. As Gittell [45] notes “Even timely, accurate information may not be heard or acted upon if the recipient does not respect the source” (p. 16).

Whilst the lack of sufficient numbers of professionals and professions available in or to rural areas impacts greatly on the capacity for IPP, there is also space for development and extension of models that involve sharing of work across disciplines. Perhaps, most significant is the need for recognition and support of pivotal roles and the processes employed by these individuals to engage others and act as a central resource for patients and their families. Additionally there is some evidence that IPP can help combat the effects of professional isolation which addresses one of the issues associated with the challenges of recruitment and retention of rural health practitioners [24]. Overall, it is evident that the processes underpinning the delivery of care are just as important as what care is delivered.

### Study strengths and limitations

A strength of the study was that data were gathered across a range of professionals, settings and contexts. A number of references to Practice Nurses by participants highlighted that inclusion of Practice Nurses’ perspective and understanding of how they contribute to rural IPP would have informed a more comprehensive understanding of contemporary primary rural health care. Further, a more holistic perspective would be gained by inclusion of patients reports of their experiences with various health professionals.

## Conclusion

Findings suggest that the nature of IPP in rural contexts is diverse and determined by a number of critical factors including rurality, connection to community, availability of staff, funding programs and specific interests and skills of staff. Most rural health professionals in our study appear motivated to engage in IPP. However, optimal outcomes of IPP may be hampered by adherence to historically embedded cultural behaviours, together with persistence of models of care that perpetuate rigid professional boundaries. This study goes some of the way towards unravelling the complexity of IPP in rural context, highlighting the strong motivating factors that drive IPP. However, it has also identified significant structural and relational barriers related to workload, workforce and service fragmentation. Further research is required to explicate the mechanisms that drive successful IPP across a range of diverse rural contexts in order to inform the implementation of robust flexible strategies that will support sustainable models of rural IPP.

## Abbreviations

AHP: Allied health practitioner; CNC: Clinical nurse consultant; GP: General practitioner; HSM: Health service manager; IPP: Interprofessional practice; MO: Medical officer; MPS: Multipurpose service centre; NSW: New South Wales; RN: Registered nurse.

## Competing interests

The authors declare that they have no competing interests.

## Authors’ contributions

VP, KM, IH, RM, PP, MG, GP were involved in the conception, design and acquisition of data, analysis and interpretation. VP & KM drafted and revised the manuscript. IH, RM, PP, MG, GP revised paper for intellectual content. All authors read and approved the final manuscript.

## Pre-publication history

The pre-publication history for this paper can be accessed here:

http://www.biomedcentral.com/1472-6963/13/500/prepub
